# LncRNA34977 promotes the proliferation, migration, and invasion and inhibits the apoptosis of canine mammary tumors by regulating the expression of miR-8881/ELAVL4

**DOI:** 10.1007/s10142-022-00955-4

**Published:** 2023-01-06

**Authors:** Baochun Lu, Yufan Zhu, Juye Wu, Huidan Qiu, Jinyu Wang, Zihang Ma, Kun Jia

**Affiliations:** 1 College of Veterinary Medicine, South China Agricultural University, Guangzhou, 510642 China; 2 Guangdong Provincial Key Laboratory of Prevention and Control for Severe Clinical Animal Diseases, Guangzhou, 510642 China; 3 Guangdong Technological Engineering Research Center for Pet, Guangzhou, 510642 China

**Keywords:** Canine mammary tumor, lncRNA34977, miRNA8881, ELAVL4, Regulation

## Abstract

Long-stranded noncoding RNAs (lncRNAs) play different roles in various diseases. lncRNA34977 has been shown to play a relevant role the development of canine mammary tumors (CMTs). However, the mechanism of lncRNA34977 in canine mammary tumors has not been fully investigated. The aim of this study was to investigate the effects of lncRNA34977 on the proliferation, migration, invasion, and apoptosis of canine mammary tumor (CMT) cells through the regulation of miR-8881/ELAVL4 expression. The apoptosis was detected by an in situ fluorescence assay and flow cytometry. The expression levels were analyzed by RT-qPCR. CCK-8, colony formation, wound healing, and Transwell assays were used to assess the proliferation, migration, and invasion. The expression of protein was detected by western blot. The siRNA-induced silencing of lncRNA34977 promoted the apoptosis of CHMp cells, and in overexpression of lncRNA34977, the result is the opposite. LncRNA34977 has a direct targeting relationship with miR-8881 and that miR-8881 is correlated with ELAVL4. Transfection of miR-8881 mimics inhibited the proliferation, migration, invasion, and promoted the apoptosis of CHMp cells of CHMp cells. In the transfection with miR-8881 inhibitors, the result is the opposite. Co-transfected with lncRNA34977, miR-8881, or ELAVL4, we found that lncRNA34977 could regulate the expression of miR-8881 or ELAVL4. Our study shows that lncRNA34977 promotes the proliferation, migration, and invasion and suppresses the apoptosis of CMT cells by regulating the expression of miR-8881/ELAVL4.

## Introduction 

Apart from canine skin tumors, canine mammary tumors (CMTs) are the most common tumors in the canine population (Kaszak et al. 2018; Rasotto et al. 2017). Canine mammary tumors account for approximately 30% of all canine tumors, with 25–42% developing in females and 70% developing in unspayed female dogs. Up to 50% of all CMTs are malignant (王靖媛, 吴乔兴, 佘锐萍, 李恒, 石蕊寒, 杨金玲, 郝文卓, 安俊卿, 苑雪瑞, 胡凤姣.(2017).^44^例宠物犬肿瘤病理诊断及分析.中国畜牧兽医, 44(03), 935–940. 2017). CMTs do not have a very uniform etiology and are thought to have a heterogeneous pathogenesis. Genetics, age, diet, environment and sterilization and other conditions caused by gonadal axis secretion disorder are common causative factors(Rasotto et al. 2017; 刘韵佳, 庞博, 李妍, 黄坚.(2021).^68^例犬乳腺肿瘤病例的回顾性分析. 西南民族大学学报(自然科学版), 47(06), 574–580. 2021;). With the progressive study of genes in humans, the occurrence of oncological diseases and abnormal gene expression have been found to be highly correlated. Noncoding RNAs (ncRNAs) can regulate gene expression at the transcriptional and posttranscriptional levels, regulate chromatin remodeling and signal transduction, and act as key regulators of cellular processes in physiological and pathological contexts. In particular, ncRNAs are closely associated with cancer and have been identified that can both inhibit the development of certain tumors and promote the development of certain tumors (Anastasiadou et al. 2018).

Long noncoding RNAs (lncRNAs) are a family of ncRNAs that are 200–100,000 nucleotides in length (Bridges et al. 2021; Xing et al. 2021). LncRNAs are closely correlated with tumors, and their abnormal expression can affect the proliferation, metastasis, invasion, and apoptosis of tumor cells and plays an important role in tumor development (Tang et al. 2019). For example, increasing evidence indicates that HOX transcript antisense RNA (HOTAIR), which is often overexpressed in a variety of cancer cells, such as colorectal, breast, and liver cancer cells, regulates cellular pathways associated with cancer cell proliferation, migration, invasion, and angiogenesis and exerts procancer effects (Yu and Li 2015). LncRNA34977 is highly expressed in canine mammary tumors and promotes the development of canine mammary tumors (Lu et al. 2022), but its regulatory mechanism in canine mammary tumors is unclear.

MicroRNAs (miRNAs), a class of small RNAs approximately 20–24 nucleotides in length, represent another type of ncRNA (Nicoloso et al. 2009; Wang et al. 2016). Increasing evidence indicates that lncRNAs can bind to miRNAs to regulate their function, which has been described as sponge regulation (Nicoloso et al. 2009). This relationship between miRNAs and lncRNAs implies that miRNAs are also closely associated with tumors. LINCHCP5 can act as an endogenous competing RNA that binds to miR-27a-3p to regulate IGF-1 protein expression, forming a lncRNA–miRNA–mRNA regulatory network that affects the proliferation, migration, invasion, and apoptosis of cancer cells (Chen et al. 2020). MiR-8881 is a member of the miRNA family and has the physiological functions that miRNAs have, but it is poorly studied in human medicine or veterinary medicine and its mechanism of action is unknown. Related proteins can be produced by binding to miRNAs, which in turn can have certain effects on various physiological processes. mRNA ELAVL4 is a segment of protein that can interact with miRNAs, and its binding to miRNAs can have effects on a range of physiological activities, but it has rarely been reported in veterinary clinical tumors, and the mechanism of action is unknown. The main objective of this study was to assess the potential impacts of lncRNA34977 on the development of canine mammary tumors by regulating the expression of miR-8881/ELAVL4 and thus provides a new avenue for the treatment of canine mammary tumors.

## Materials and methods

### Sample collection and processing

Tumor samples and nonneoplastic tissues samples (2 cm from the tumor tissue) of 30 canine mammary tumors were obtained from various animal hospitals in Guangzhou, China, and none of the 30 bitches had received any treatment or medication. These 30 specimens were grade III (G3) poorly differentiated malignant tumors. We followed the animal welfare policy and obtained the owner’s consent. We stored the samples at − 80 °C after processing. Samples were extracted for total RNA and then used for RT-qPCR experiments.

### Cell culture and transfection

A stable CMT cell lines (CHMp) were used in this study, provided by the Laboratory of Veterinary Surgery, Department of Agronomy, Faculty of Life Sciences, University of Tokyo, Japan. Cells were cultured in DMEM, addition of 1% penicillin–streptomycin solution (100X) (C0222, Beyotime, Shanghai, China) and 10% Australian fetal bovine serum (Thermo Fisher Scientific, Waltham, MA, USA). Place the cells in the incubator; the incubator (Thermo Fisher Scientific, Waltham, MA, USA) was maintained at 37 °C and 5% CO_2_. Transfection was performed according to Lipofectamine 3000 reagent, and plate cells were transfected at a concentration of 5 × 105 in six-well plates. MiRNA transfection was performed as described for siRNAs. LncRNA34977 siRNA and a negative control (NC), miR-8881 mimics and a miR-8881 mimic NC, an miR-8881 inhibitor and a miR-8881 inhibitor NC, and ELAVL4 siRNA and a negative control (NC) were all purchased from Shanghai GenePharma Inc. (Shanghai, China). The p3FLAG-N-CMV-10-lncRNA34977 lncRNA34977 overexpression plasmid was constructed using the p3FLAG-N-CMV-10 vector. The sense and antisense siRNA lncRNA34977 sequences were 5′-CCCGGGAUACUGCCUACAUTT-3′ and 5′-AUGUAGGCAGUAUCCCGGGTT-3′. The sense and antisense siRNA lncRNA 34,977 NC sequences were 5′-UUCUCCGAACGUGUCACGUTT-3′ and 5′-ACGUGACACGUUCGGAGAATT-3′. The sense and antisense siRNA ELAVL4 sequences were 5′-GCAGUAGGUGCUACGAAAUTT-3′ and 5′-AUUUCGUAGCACCUACUGCTT-3′. The sense and antisense siRNA ELAVL4 NC sequences were 5′-UUCUCCGAACGUGUCACGUTT-3′ and 5′-ACG UGACACGUUCGGAGAATT-3′. The sense and antisense miR-8881 mimic sequences were 5′-UUUGUUUUCUCUGGUUCUGUACC-3′ and 5′-UACAGAACCAGAGAAAACAAAUU-3′. The sense and antisense miR-8881 mimic NC sequences were 5′-UUCUCCGAACGUGUCACGUTT-3′ and 5′-ACGUGACACGUUCGGAGAATT-3′. The sense and antisense miR-8881 inhibitor and inhibitor NC sequences were 5′-GGUACAGAACCAGAGAAAACAAA-3′ and 5′-CAGUACUUUUGUGUAGUACAA-3′.

To verify the role of miR-8881 in CHMp cells, the miR-8881 mimic or mimic NC and the miR-8881 inhibitor or miR-8881 inhibitor NC were transfected into CHMp cells with Lipofectamine 3000 (Thermo Fisher Scientific, Waltham, MA, USA) and cultured for 48 h. To verify that lncRNA34977 affects the function of CHMp cells through miR-8881, lncRNA34977 siRNA or the NC was transfected into CHMp cells with the miR-8881 inhibitor or inhibitor NC and cultured for 48 h. ELAVL4 siRNA or NC was transfected into CHMp cells with miR-8881 mimics cultured for 48 h.

### RT-qPCR

After extracting the total RNA, we detected the OD 260/280 values of RNA, which were used to calculate the RNA concentration. The RNA was reversed to cDNA according to HiScript II Q RT SuperMix for qPCR (Vazyme Biotech, Nanjing, China); the cDNA was premixed with ChamQ Universal SYBR qPCR Master Mix (Vazyme Biotech, Nanjing, China) for quantitative real-time polymerase chain reaction. ACTB was used as an internal control group. The sense and antisense primers encoding miR-8881, ELAVL4, Bcl-2, and Bax were designed using Vazyme miRNA primer design software (Vazyme Biotech, Nanjing, China) and Primer Premier 6.0 (PREMIER Biosoft, San Francisco, CA, USA) software as follows:

miR-8881 sense primer, 5′-GGTTTCAAAGATAGAGCTGGTAAGTTT-3′

miR-8881 antisense primer, 5′-AGTGCAGGGTCCGAGGTATT-3′

ELAVL4 sense primer, 5′-ATAGCGATGTGCAGGGAT-3′

ELAVL4 antisense primer, 5′-TCAAATGAGCGAATGGTC-3′

Bax sense primer, 5′-CGCAGATGAACTGGACAGTAAC-3′

Bax antisense primer, 5′-AAGTAGAAGAGGGCAACAACC-3′

Bcl-2 sense primer, 5′-CTTCGCCGTGATGTCCAG-3′

Bcl-2 antisense primer, 5′-CAGATGCGGGTTCAGATAC-3′

ACTB sense primer, 5′-GGCATCCTGACCCTGAAGTA -3′

ACTB antisense primer, 5′-GGGGTGTTGAAAGTCTCGAA-3′.

Collating the average CT values (amplified power curve inflection point) of each group, the melting curve of this experiment can be referred to in order to ensure the accuracy and reliability of the results. After the average CT values were obtained, the relative gene expression levels were calculated by 2^−△△Ct^ analysis.

### Cell Counting Kit-8

The cell proliferation capacity of the cells was assayed using Cell Counting Kit-8 (CCK-8; Beyotime Biotechnology, Shanghai, China). The transfected cells were resuspended, and 2000 cells were transferred to 96-well plates. Twenty-four hours later, 10 µL of CCK-8 reagent was added to the cells in the 96-well plates and incubated for 3.5 h. Absorbance was measured at 450 nm using a Multiskan FC (Thermo Fisher Scientific, Waltham, MA, USA) every 24 h. Repeat for 3 days.

### Clone formation

Resuspend and mix the transfected cells, aspirate 250 cells to transfer into a six-well plate, allow the cells to be evenly distributed, and incubate for 7–10 days. After the appearance of clones containing ≥ 20 cells, the cells were fixed with 95% ethanol for 20 min, PBS was rinsed twice, then stained with 10% Giemsa dye solution for 20 min. After taking photographs, record the experimental data; (clone number/number of inoculated cells) × 100%.

### Wound healing

After the cells have grown to 75–80% in the well plate and then scratched with 10 µl gun tip. Followed by rinsing with PBS, the cells were cultured with DMEM without FBS. Photographs were taken to record the wound healing at 0 h and 24 h.

### Cell invasion

Diluting Matrigel with DMEM in a ratio of 1:8.to make a premix. Fifty microliters of the premix was added to the Transwell insert and placed in the incubator at 37 ℃and 5% CO_2_ for 4–6 h. Two hundred microliters of cytosol at a concentration of 1 × 10^5^ was added to the Transwell insert, and 500 µl of DMEM containing 10% FBS was added to the bottom of the 24-well plate and incubated in the incubator for 24 h. After 24 h, the cells were washed twice with PBS, fixed with 100% methanol for 20 min, then washed with PBS, and stained with 10% Giemsa for 20 min. Excess cells on the vesicle membrane were removed with cotton swabs, observed under the microscope, and photographed for counting.

### Flow cytometry analysis

Cells were transfected and incubated for 48 h. Apoptotic cells were analyzed by flow cytometry (Beckman, Pasadena, CA, USA). Briefly, for apoptosis analysis, cells were collected and stained with Annexin V-FITC and propidium iodide (PI) in the dark according to the procedure of the Apoptosis Detection Kit (C1062S, Beyotime, Shanghai, China). The cells were incubated for 15–20 min at room temperature (20–25 ℃) in the dark and subsequently placed in an ice bath. Flow cytometry was then performed.

### In situ* fluorescence detection*

Cells were transfected and incubated for 48 h. Apoptotic cells were analyzed by fluorescence microscopy. Briefly, cells were stained with the membrane-linked protein V-FITC and propidium iodide (PI) in the dark according to the procedure of the Apoptosis Detection Kit (C1062S, Beyotime, Shanghai, China), incubated for 20 min at room temperature (25 ℃) protected from light, and immediately after the end and placed in an ice bath.The cells were immediately observed under a fluorescence microscope, with Annexin V-FITC being indicated by green fluorescence and propidium iodide (PI) being indicated as red fluorescence.

### Western blot

After transfecting CHMp cells with the miR-8881 mimic or mimic NC, or the miR-888 inhibitor or inhibitor NC for 48 h, the cells were lysed with a protein extraction kit (P0013, Beyotime, Shanghai, China) in accordance. The total protein of the desired sample was extracted using the method of this kit. The sample protein concentrations were measured by the BCA protein concentration assay kit (P0010S, Beyotime, Shanghai, China). Calculated the amount of protein according to the protein amount. Same amount of protein were separated by SDS–PAGE on a 12.5% gel and electroblotted onto polyvinylidene fluoride membranes (IPVH00010, Millipore, Massachusetts, USA). The membranes were blocked with Protein-free Rapid Sealer 5x (PS108, Epizyme, Shanghai, China) for 10–15 min and incubated overnight at 4 ℃ with primary antibodies against ELAVL4 (1:500, A10655, ABclonal, Wuhan, China) and GAPDH (1:1000, AF0006, Beyotime, Shanghai, China). After washing the membrane twice with PBST buffer for 5 min each time, the membranes were incubated with a horseradish peroxidase-conjugated secondary antibody for 1–2 h at 37 ℃.

### Dual-luciferase reporter assay

Wild-type (WT) and mutant (Mut) reporter plasmids for lncRNA34977 and ELAVL4-3’UTR were constructed; lncRNA34977-Mut and ELAVL4-3’UTR-Mut were obtained from Gene Create (Wuhan, China) and Sangon Biotech (Shanghai, China), respectively. For the luciferase reporter assay, CHMp cells were spread in 24-well plates in DMEM supplemented with 10% FBS and placed in a cell culture incubator. The lncRNA34977 (WT and Mut) or ELAVL4-3’ UTR (WT and Mut) was cotransfected with the miR-8881 mimic or mimic NC using Lipofectamine 3000. Forty-eight hours after transfection, the cells were lysed, and the cell lysates were subjected to a dual luciferase assay using the Dual Luciferase Reporter Gene Assay Kit (RG027, Beyotime, Shanghai, China). The results are expressed as relative luciferase activity (Firefly luciferase/Renilla luciferase).

### Statistical analysis

The experimental data were statistically analyzed using GraphPad Prism software version 6.0 (GraphPad Software Inc.), and the results were calculated using statistical software 19.0 (IBM, New York, NY, USA). Data from three and more groups were analyzed using one-way ANOVA, while data from two groups were analyzed using *t*-test. All experimental groups were repeated three times to ensure the reliability of the experimental data, and the data obtained were the mean ± standard deviation. The results were statistically significant and significantly different when *p* < 0.05 and highly statistically significant when *p* < 0.01.

## Results

### LncRNA34977 inhibits the apoptosis of CMT cells

**Fig. 1:**
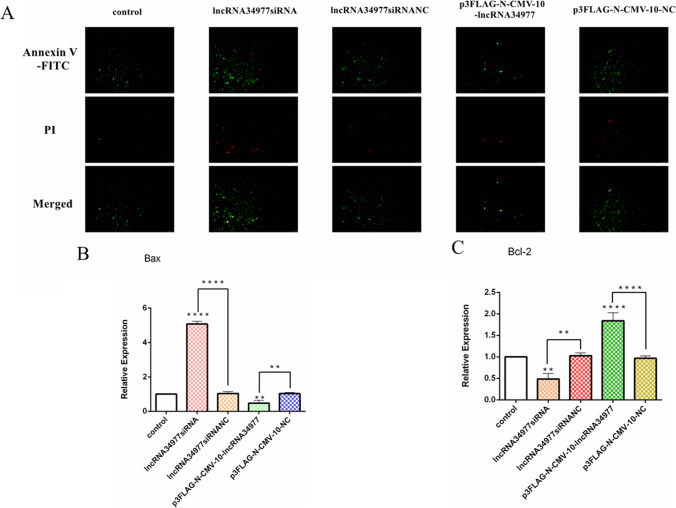
LncRNA34977 inhibits the apoptosis of CMT cells. **A** Effect of lncRNA34977 on the apoptosis of CMT cells. **B**, **C** After transfection of lncRNA34977 siRNA or NC and p3FLAG-N-CMV-10-lncRNA34977 or p3FLAG-N-CMV-10-NC, the expression levels of Bax and Bcl-2 were determined by RT-qPCR (*P < 0.05, **P < 0.01, ***P < 0.001, ****P < 0.0001)

To investigate the effect of lncRNA34977 on the apoptosis of CHMp cells, we transfected lncRNA siRNA and lncRNA siRNA NC or p3FLAG-N-CMV-10-lncRNA34977 and p3FLAG-N-CMV-10-lncRNA34977 NC into cells and then detected apoptosis by an in situ fluorescence assay. The results showed that transfection of the lncRNA siRNA promoted apoptosis and that overexpression of lncRNA34977 inhibited apoptosis (Fig. 1A). We also detected the expression levels of Bax and Bcl-2 in the different transfection groups by RT-qPCR (Fig. 1B, C), and the results showed that Bax expression was upregulated in the cells transfected with the lncRNA siRNA and downregulated in cells overexpressing lncRNA34977; Bcl-2 expression was upregulated in cells overexpressing lncRNA34977 and downregulated in cells with siRNA-induced lncRNA silencing.

### Prediction and confirmation of the direct roles of lncRNA34977 and miR-8881

**Fig. 2:**
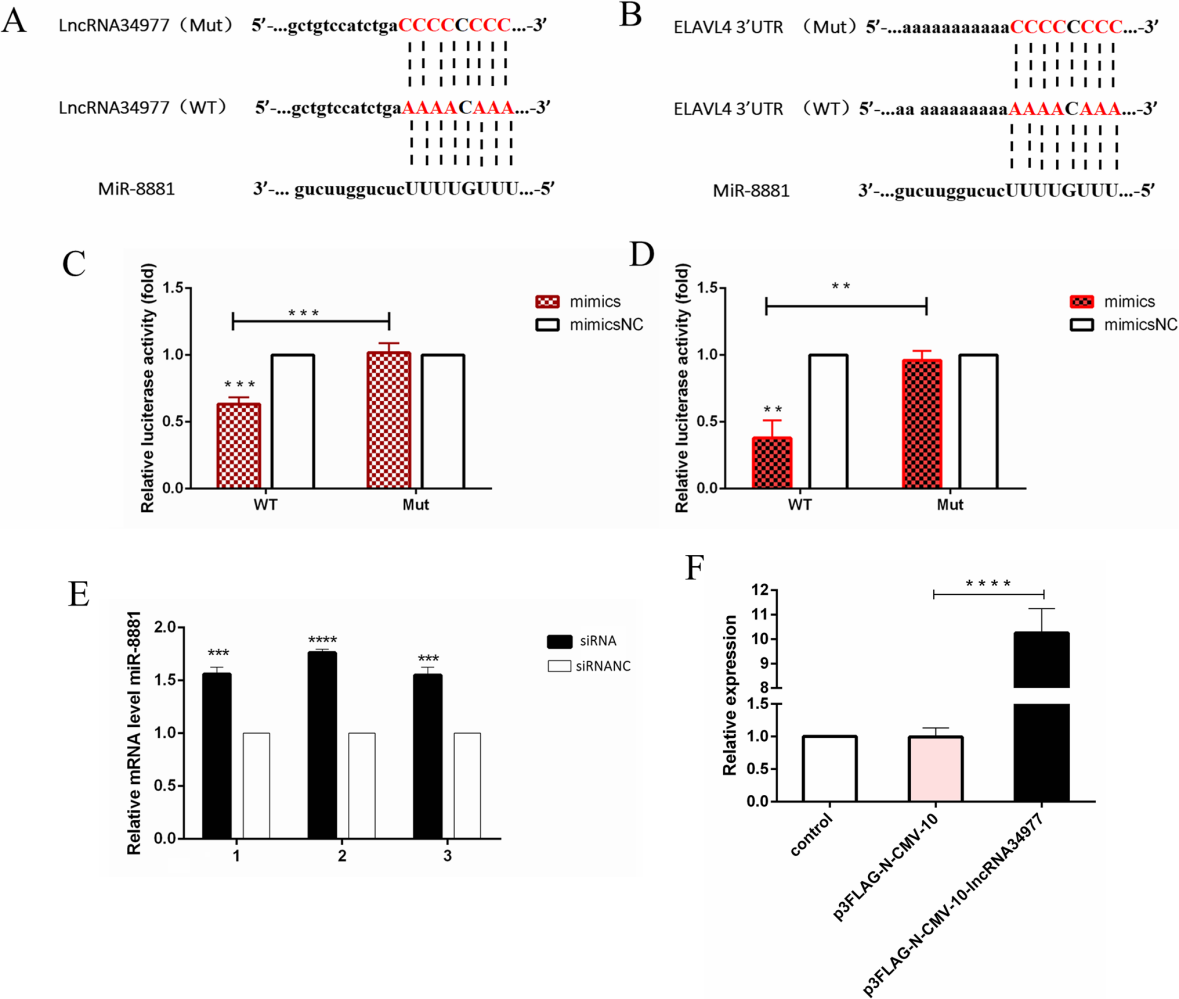
Prediction and confirmation of the direct roles of lncRNA34977 and miR-881. **A**, **B** The predicted binding sites of miR-8881 on lncRNA34977 and ELAVL4. The wild-type or mutant lncRNA34977 (lncRNA34977 WT and lncRNA34977 Mut) and the ELAVL4-3′ UTR (ELAVL4 WT and ELAVL4 Mut) were cotransfected with the miR-8881 mimic or negative control (mimic NC). **C**, **D** Luciferase activities were detected 48 h after transfection. **E** Expression levels of miR-8881 in CHMp cells transfected with the siRNA or siRNA NC. **F** Expression levels of lncRNA34977 in CHMp cells transfected with the p3FLAG-N-CMV-10-lncRNA34977 or p3FLAG-N-CMV-10 NC (*P < 0.05, **P < 0.01, ***P < 0.001, ****P < 0.0001)

We examined the relative expression of overexpressed lncRNA34977, and the results showed that the expression of lncRNA34977 was significantly increased in the overexpression group compared with the blank group and the control group (Fig. 2F). We predicted the direct targets of lncRNA34977 using the online bioinformatics tool TargetScan. We found that lncRNA34977 has binding sites for miR-8881 (Fig. 2A). We predicted that ELAVL4 was a potential target of miR-8881 (Fig. 2B). Subsequently, we constructed wild-type (WT) and mutant (Mut) luciferase reporter vectors for lncRNA34977 and ELAVL4-3’ UTR using the psiCHECK2 plasmid. Then, the cells were cotransfected with the miR-8881 mimic or mimic NC. The luciferase activities were measured 48 h after transfection. The miR-8881 mimic significantly attenuated the luciferase activity in cells transfected with the lncRNA34977-WT or ELAVL4-3’ UTR-WT plasmid, but the luciferase activity was not altered in cells transfected with the lncRNA34977-Mut or ELAVL4-3’ UTR-Mut plasmids (Fig. 2C, D). The results of this experiment indicate that miR-8881 can bind to the 3′ UTR of the lncRNA34977 gene and inhibit its luciferase activity and bind to the ELAVL4-3′ UTR and inhibit its luciferase activity. To further confirm the relationship between lncRNA34977 and miR-8881, we also transfected an siRNA targeting lncRNA34977 into CHMp cells and assessed miR-8881 expression by RT-qPCR after 24 h. The experiments were repeated three times, and the results showed that miR-8881 expression was upregulated in CHMp cells treated with the lncRNA34977 siRNA (Fig. 2E). In summary, miR-8881 is a downstream target of lncRNA34977, and the two are inversely regulated. ELAVL4 is a downstream target of miR-8881.

### Both miR-8881 and ELAVL4 are expressed at low levels in CMT and are positively regulated

**Fig. 3:**
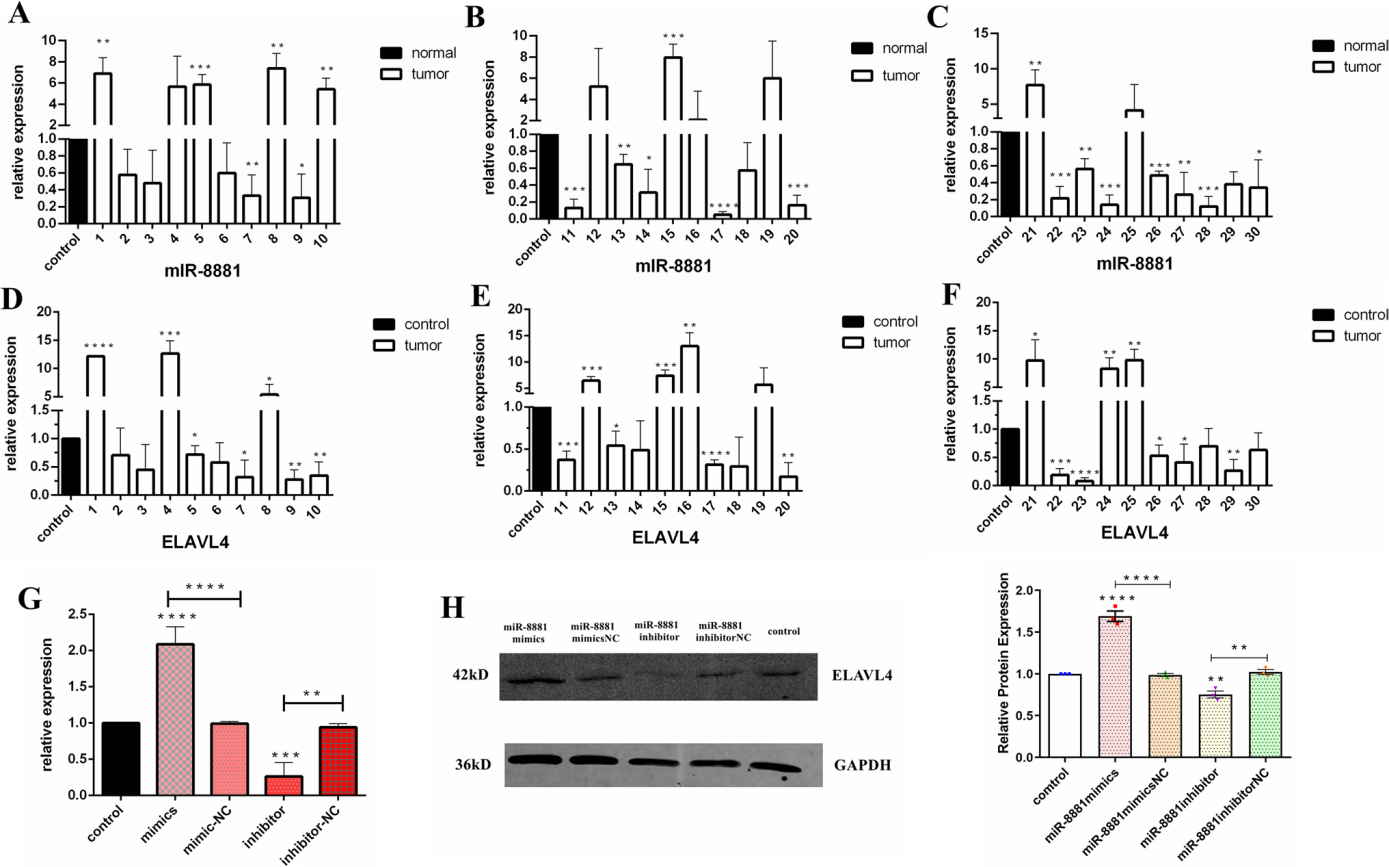
miR-8881 and ELAVL4 are expressed at low levels in CMT cells and positively regulated. **A**–**C** The expression levels of miR-8881 in CMT and adjacent normal tissues as determined measured by RT-qPCR. Samples 1–30 (*P < 0.05, **P < 0.01, ***P < 0.001, ****P < 0.0001). **D**–**F** The expression levels of ELAVL4 in CMT and adjacent normal tissues as measured by qRT-PCR. Samples 1–30. **G** After transfection of the miR-8881 mimic or mimic NC and the miR-8881 inhibitor or inhibitor NC, the expression levels of ELAVL4 in CMT and adjacent normal tissues were measured by RT-qPCR. **H** Expression of ELAVL4 in CHMp cells transfected with the miR-8881 mimic or mimics NC and the miR-8881 inhibitor or miR-8881 inhibitor NC (*P < 0.05, **P < 0.01, ***P < 0.001, ****P < 0.0001)

To investigate the functions of miR-8881 and ELAVL4 in CMT, their expression in CMT tissues was detected by RT-qPCR. The results revealed distinct downregulation of the miR-8881 and ELAVLV4 levels in CMT tissues compared to adjacent normal tissues (Fig. 3A, B, C). To verify the RT-qPCR results regarding miR-8881 with ELAVL4, we transfected the miR-8881 mimic or mimic NC and the miR-8881 inhibitor or inhibitor NC, and total RNA was extracted after 48 h and subjected to RT-qPCR. The results showed that ELAVL4 expression was upregulated in cells transfected with the miR-8881 mimic compared with those transfected with the miR-8881 mimic NC (Fig. 3D, E, F); ELAVL4 expression was downregulated in the cells transfected with the miR-8881 inhibitor compared with those transfected with the miR-8881 inhibitor NC (Fig. 3G). The Western blot results showed that ELAVL4 expression was upregulated in the cells transfected with the miR-8881 mimic and downregulated in those transfected with the miR-8881 inhibitor (Fig. 3H). In summary, miR-8881 and ELAVL4 are expressed at low levels in CMT and are positively regulated by each other.

### Effect of miR-8881 on the proliferation, migration, invasion, and apoptosis of CMT cells

**Fig. 4:**
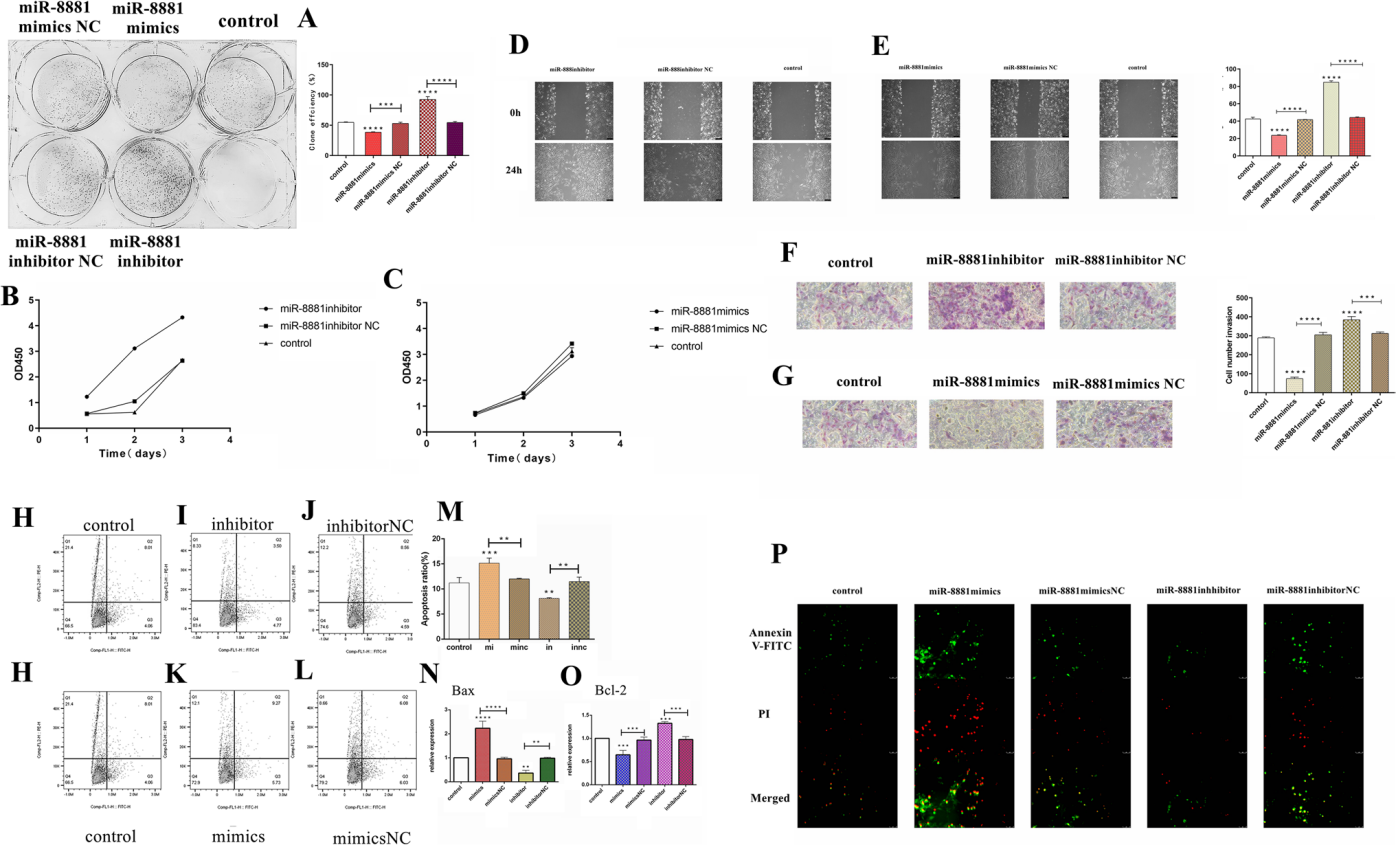
Effect of miR-8881 on the proliferation, migration, invasion and apoptosis of CMT cells. **A** The miR-8881 mimic and inhibitor the colony formation assay (*P < 0.05, **P < 0.01, ***P < 0.001, ****P < 0.0001). (**B**) Cell Counting Kit-8 cell growth curve of CHMp cells transfected with the miR-8881 inhibitor or inhibitor NC. **C** Cell Counting Kit-8 cell growth curve of CHMp cells transfected with the miR-8881 mimic—or mimic NC. **D** Effects of the miR-8881 inhibitor and inhibitor NC on the migration of CHMp cells. **E** Effects of the miR-8881 mimic and mimic NC on the migration of CHMp cells. **F** Effects of the miR-8881 inhibitor and inhibitor NC on the invasion of CHMp cells. **G** Effects of the miR-8881 mimic (mi) and mimic NC on the invasion of CHMp cells (*P < 0.05, **P < 0.01, ***P < 0.001, ****P < 0.0001). **H**–**J** Effects of the miR-8881 inhibitor and inhibitor NC on the apoptosis of CHMp cells as detected by flow cytometry. (**M**) Flow cytometry analysis of the effect of miR-8881 on the apoptosis of CHMp cells. **H**, **K**, **L** Effect of the miR-8881 mimic or mimic NC on the apoptosis of CHMp cells as detected by flow cytometry. **N**, **O** After transfection of the miR-8881 mimic or mimic NC and the miR-8881 inhibitor or inhibitor NC, the expression levels of Bax and Bcl-2 were determined by RT-qPCR (*P < 0.05, **P < 0.01, ***P < 0.001, ****P < 0.0001). **P** Effect of miR-8881 on the apoptosis of CMT cells. The green fluorescence indicates Annexin V-FITC-positive cells, and the red fluorescence indicates propidium iodide-positive cells. Apoptotic cells are stained by green fluorescence only, necrotic cells are stained by both green and red fluorescence, and normal cells are not fluorescent

We transfected CHMp cells with the miR-8881 mimic or mimic NC and the miR-8881 inhibitor or inhibitor NC. To investigate the effect of miR-8881 on the proliferation of CHMp cells, we performed Cell Counting Kit-8 and colony formation assays, and the results showed that the miR-8881 mimic inhibited the proliferation of CHMp cells (Fig. 4A, C). In contrast, the miR-8881 inhibitor promoted the proliferation of CHMp cells (Fig. 4A, B). To assess the effect of miR-8881 on CHMp cell migration and invasion, we performed wound healing and cell invasion assays, which showed that the miR-8881 mimic inhibited CHMp cell migration (Fig. 4E) and invasion (Fig. 4G) and that the miR-8881 inhibitor promoted CHMp cell migration (Fig. 4D) and invasion (Fig. 4F). To verify the effect of miR-8881 on the apoptosis of CHMp cells, we performed RT-qPCR, in situ fluorescence and flow cytometry analyses, and the RT-qPCR results showed that Bax was highly expressed in cells transfected with the miR-8881 mimic (Fig. 4N) and expressed at a low level in cells transfected with the miR-8881inhibitor (Fig. 4N); Bcl-2 was highly expressed in cells transfected with the miR-8881 inhibitor (Fig. 4O) and expressed at low levels in cells transfected with the miR-8881 mimics (Fig. 4O). The results together with those of flow cytometry analysis (Fig. 4M) demonstrate that the miR-8881 mimic promoted the apoptosis of CHMp cells (Fig. 4K, L), while the miR-8881 inhibitor inhibited apoptosis (Fig. 4I, J). The results of the in situ fluorescence assay were consistent with those of flowcytometry (Fig. 4P).

### LncRNA34977 regulated the expression of miR-8881

**Fig. 5:**
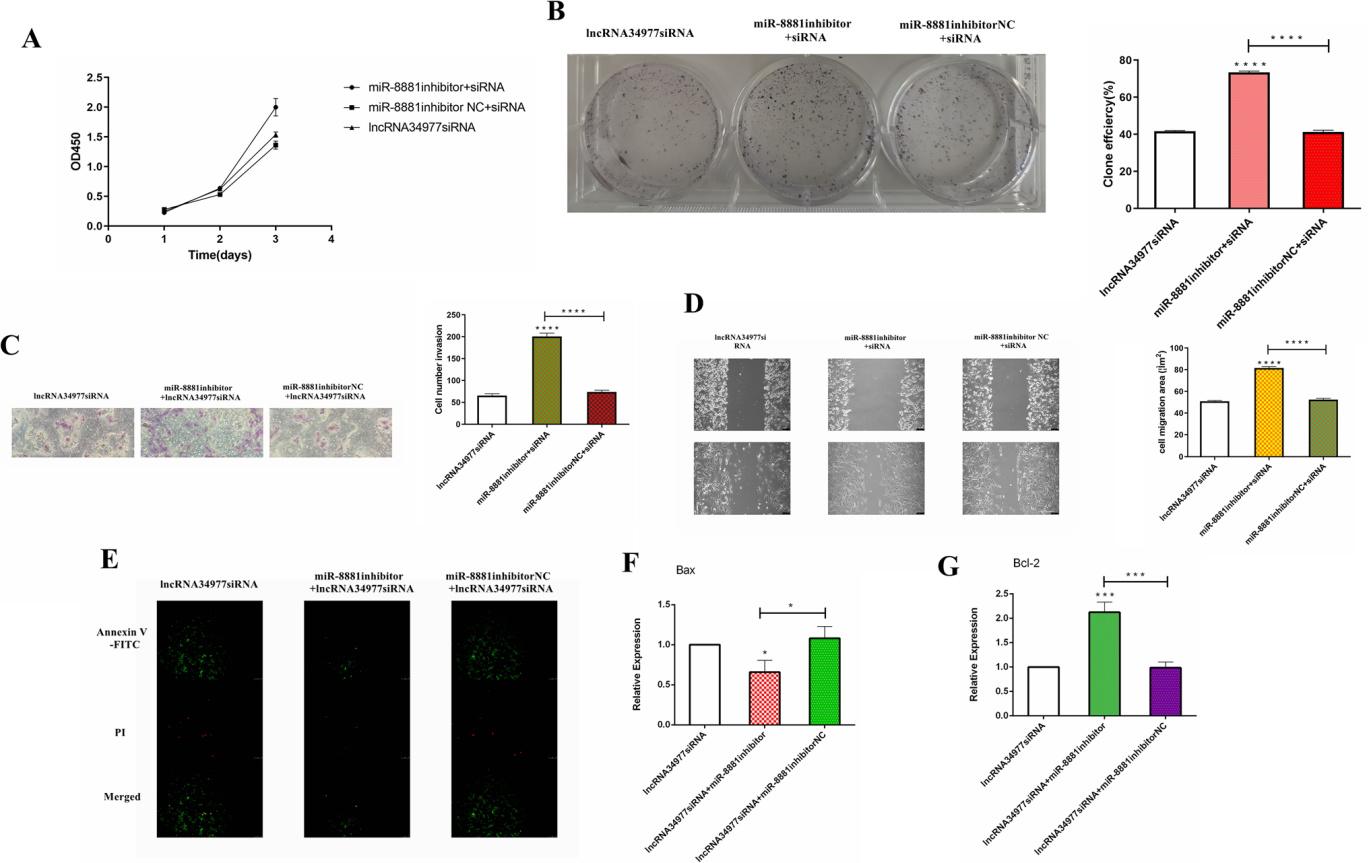
LncRNA34977 regulated the expressions of miR-8881. **A** Experimental growth curve of CHMp cells contransfected with the lncRNA34977 siRNA and miR-8881 inhibitor or inhibitor NC as determined by the CCK-8 assay. **B** Colony formation assay of CHMp cells contransfected with the lncRNA34977 siRNA and miR-8881 inhibitor or inhibitor NC. **C** Effect of contransfecting the lncRNA34977 siRNA and miR-8881 inhibitor or inhibitor NC on CHMp cell invasion. **D** Wound healing assay of CHMp cells contransfected with the lncRNA34977 siRNA and the miR-8881 inhibitor or inhibitor NC (*P < 0.05, **P < 0.01, ***P < 0.001, ****P < 0.0001). **E** Effect of contransfecting the lncRNA34977 siRNA and the miR-8881 inhibitor or inhibitor NC on the apoptosis of CMT cells. The green fluorescence indicates Annexin V-FITC-positive cells, and the red fluorescence indicates propidium iodide-positive cells. Apoptotic cells are stained by green fluorescence only, necrotic cells are stained by both green and red fluorescence, and normal cells are not fluorescent. **F**, **G** After cotransfection of the lncRNA34977 siRNA with the miR-8881 inhibitor or inhibitor NC, the expression levels of Bax and Bcl-2 were detected by RT-qPCR (*P < 0.05, **P < 0.01, ***P < 0.001, ****P < 0.0001)

To investigate the potential regulatory mechanism of lncRNA34977 in CHMp cells, we investigated its potential ability to regulate miR-8881 expression. We cotransfected lncRNA34977 siRNA with the miR-8881 inhibitor or inhibitor NC into CHMp cells and then performed the Cell Counting Kit-8 (Fig. 5A), colony formation (Fig. 5B), wound healing (Fig. 5D), cell invasion (Fig. 5C), and in situ fluorescence (Fig. 5E) assays to investigate the effects of knocking down lncRNA34977 and miR-8881 on the proliferation, migration, invasion, and apoptosis of CHMp cells. We also detected the expression levels of Bax and Bcl-2 in the different transfection groups by RT-qPCR (Fig. 5F, G). Cotransfection of the lncRNA34977 siRNA and miR-8881 inhibitor promoted the proliferation, migration, and invasion of CHMp cells and tended to inhibit apoptosis, and the miR-8881 inhibitor reversed the inhibitory effect of silencing lncRNA34977 on the proliferation, migration, and invasion of CHMp cells and promoted apoptosis.

### MiR-8881 regulates the expression of ELAVL4

**Fig. 6:**
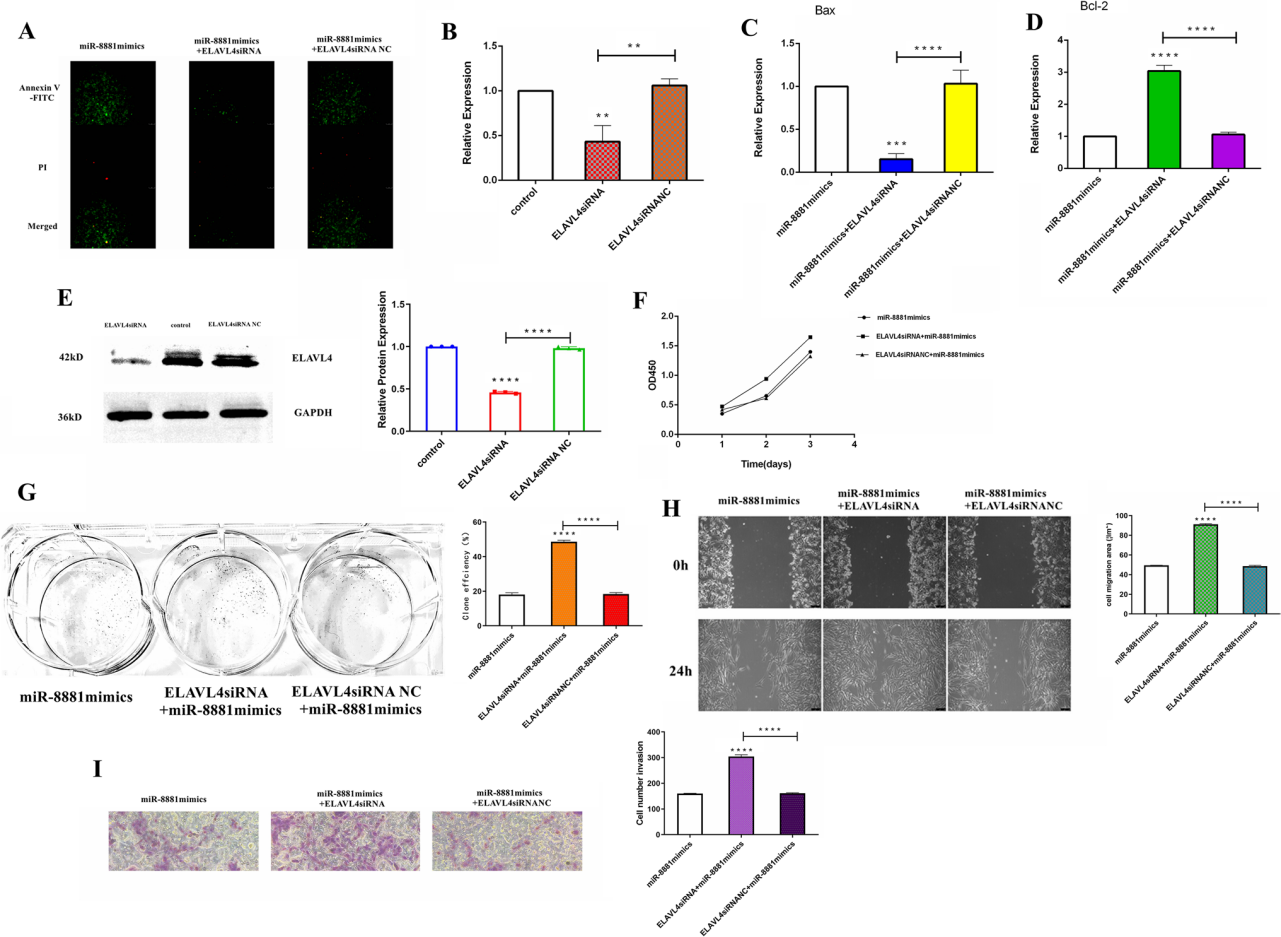
MiR-8881 regulates the expression of ELAVL4. **A** Effect of contransfecting the miR-8881 mimic with the ELAVL4 siRNA or ELAVL4 siRNA NC on the apoptosis of CMT cells. The green fluorescence indicates Annexin V-FITC-positive cells, and the red fluorescence indicates propidium iodide-positive cells. Apoptotic cells are stained by green fluorescence only, necrotic cells are stained by both green and red fluorescence, and normal cells are not fluorescent. **B** After the transfection of ELAVL4 siRNA or ELAVL4 siRNA NC, the expression levels of ELAVL4 were measured by qRT-PCR. **C**, **D** After transfection of ELAVL4 siRNA or ELAVL4 siRNA NC, the expression levels of Bax and Bcl-2 were measured by RT-qPCR. **E** After the transfection of ELAVL4 siRNA or ELAVL4 siRNA NC, the expression levels of ELAVL4 were measured by Western blot (*P < 0.05, **P < 0.01, ***P < 0.001, ****P < 0.0001). **F** Experimental growth curve of CHMp cells cotransfected with the miR-8881 mimic and ELAVL4 siRNA or ELAVL4 siRNA NC as determined by the CCK-8 assay. **G** Colony formation assay of CHMp cells cotransfected with the miR-8881 mimic and ELAVL4 siRNA or ELAVL4 siRNA NC. **H** Wound healing assay of CHMp cells cotransfected with the miR-8881 mimic and ELAVL4 siRNA or ELAVL4 siRNA NC. **I** Effect of the cotransfection of the miR-8881 mimic and ELAVL4 siRNA or ELAVL4 siRNA NC on CHMp cell invasion (*P < 0.05, **P < 0.01, ***P < 0.001, ****P < 0.0001)

To verify the regulatory effect of ELAVL4 on miR-8881, ELAVL4 siRNA was first transfected into CHMp cells, and ELAVL4 expression was then detected by RT-qPCR and Western blotting. The results showed that ELAVL4 expression was downregulated in both ELAVL4 siRNA groups (Fig. 6B, E). Then, we cotransfected ELAVL4 siRNA or the ELAVL4 siRNA NC with the miR-8881 mimic into CHMp cells and then performed the cell counting kit-8, colony formation, wound healing, cell invasion, and in situ fluorescence assays to investigate the effects of cotransfecting the ELAVL4 siRNA with the miR-8881 mimic on the proliferation, migration, invasion and apoptosis of CHMp cells. We also detected the expression levels of Bax and Bcl-2 in the different transfection groups by RT-qPCR (Fig. 6C, D). The cotransfection of ELAVL4 siRNA and the miR-8881 mimic promoted the proliferation (Fig. 6F, G), migration (Fig. 6H), and invasion (Fig. 6I) of CHMp cells and tended to reduce apoptosis (Fig. 6A), and silencing ELAVL4 reversed the inhibitory effect of the miR-8881 mimic on the proliferation, migration and invasion of CHMp cells and promoted apoptosis.

### LncRNA34977 regulated the expression of miR-8881/ELAVL4

**Fig. 7:**
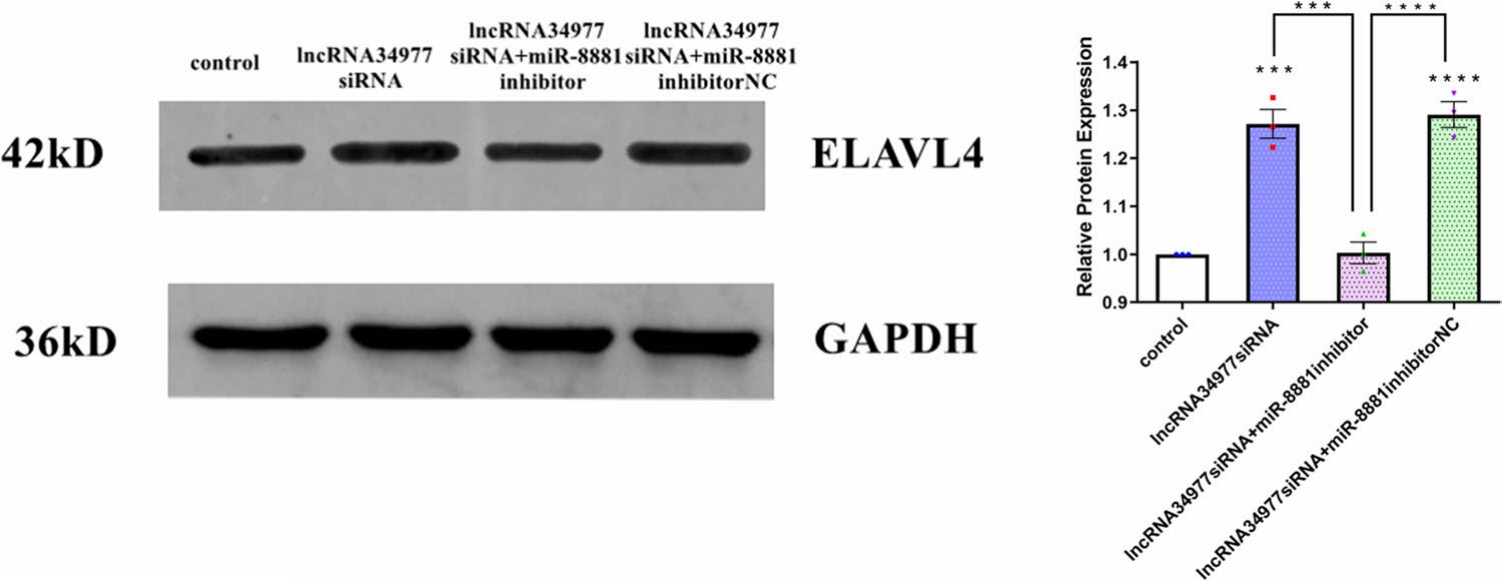
LncRNA34977 regulated the expression of miR-8881/ELAVL4 (*P < 0.05, **P < 0.01, ***P < 0.001, ****P < 0.0001)

We also cotransfected lncRNA34977siRNA lncRNA34977 siRNA with the miR-8881 inhibitor or inhibitor NC, extracted total protein after 48 h and performed a Western blot assay. The downregulation of miR-8881 reduced the increase in the ELAVL4 expression caused by the downregulation of lncRNA34977 (Fig. 7).

## Discussion

Studies have shown that many lncRNAs are aberrantly expressed in human tumors and affect tumor development (Lu et al. 2022; Zhang et al. 2022; Zhu et al. 2022; Li et al. 2022). LncRNA34977 has been shown to be highly expressed in canine mammary tumors and to promote their development. However, how lncRNA34977 regulates the development of CMT is still unknown. In this study, we demonstrated the targeting relationship between lncRNA34977 and miR-8881, and lncRNA34977 acted as a promoter in CMT cells by inversely regulating miR-8881. In addition, we also validated the role of miR-8881 in CMT cells and confirmed that miR-8881 positively regulates ELAVL4 expression and that ELAVL4 is a target of miR-8881.

LncRNAs are found in a variety of biological functions and in many diseases, such as cancer, cardiovascular diseases, and inflammatory responses. LncRNA LIFR-AS1 regulates the development of gastric cancer cells by sponging miR-4698 (Zhao et al. 2021); Yang et al. found that the lncRNA BRCAT54 can suppress tumorigenesis in non-small cell lung cancer by binding to related factors or genes (Yang et al. 2021), and some researchers found that lncRNA H19 suppresses the inflammatory response of human retinal epithelial cells by regulating related factors (Luo et al. 2021). Similarly, miRNAs, as members of the noncoding RNA family, are also involved in the regulation of many diseases (Ferragut et al. 2020; Kim and Pak 2020; Schell and Rahman 2021). miRNA-489 potentially functions as a tumor suppressor in various cancers. miRNA-489 can sensitize cancer cells to chemotherapy by disrupting molecular pathways involved in cancer growth (e.g., PI3K/Akt) and inducing apoptosis (Ferragut et al. 2020; Kim and Pak 2020; Schell and Rahman 2021; Paskeh et al. 2021). The effect of miR-8881 on canine mammary tumors was previously unknown but verified herein. We found that the miR-8881 mimic inhibited the proliferation, migration and invasion of CMT cells while promoting their apoptosis, and the miR-8881 inhibitor promoted the proliferation, migration, and invasion of CMT cells while inhibiting their apoptosis.

LncRNAs and miRNAs have relative targeting relationships in cancer, and the two influence tumor development through their interactions. Like lncRNA-RMRP, which promotes bladder cancer proliferation, migration, and invasion through miR-206(Cao et al. 2019), Peng et al. found that lncRNA PSMA3-AS1 promotes colorectal cancer cell migration and invasion by regulating miR-4429(Peng et al. 2020), and Luo et al. found that lncRNA DANCR promotes pancreatic cancer proliferation and metastasis by regulating miRNA-33b (Luo et al. 2020). In this study, we determined the targeting relationship between lncRNA34977 and miR-8881 by performing a dual luciferase assay, and the results identified miR-8881 as a downstream target of lncRNA34977. To verify its relative regulatory role, we performed CCK-8, colony counting, wound healing, Transwell, and apoptosis assays, revealing that lncRNA34977 affects canine mammary tumorigenesis by regulating miR-8881. To verify the targeting relationship between miR-8881 and ELAVL4, we performed a dual luciferase assay, identifying ELAVL4 as a downstream target gene of miR-8881. To verify its relative regulatory role, we performed CCK-8, colony counting, wound healing, Transwell, and apoptosis assays, and the results showed that miR-8881 affects canine mammary tumorigenesis by regulating ELAVL4. We also examined the expression of ELAVL4 in miR-8881-treated cells by performing the WB assay. Finally, we examined the expression of ELAVL4 in CMT cells under the action of lncRNA34977 and miR-8881 by WB, elucidating the relationship between lncRNA34977 and ELAVL4. In this study, we found that lncRNA34977 promoted the proliferation, migration and invasion of canine mammary tumors and inhibited their apoptosis by regulating the expression of miR-8881/ELAVL4.

## Data Availability

The datasets generated during and/or analyzed during the current study are available from the corresponding author on reasonable request.

## Abbreviations

CMTs: Canine mammary tumors
LncRNAs: Long noncoding RNAs
LncRNA34977: Long noncoding RNA 34,977
MiRNAs: MicroRNAs
MiR-8881: MicroRNA8881
RT-qPCR: Real-time quantitative PCR
CCK-8: Cell counting kit-8
WT: Wild-type
Mut: Mutant
NC: Non-specific control
